# Solid‐State Structure of Tris‐Cyclopentadienide Uranium(III) and Plutonium(III)

**DOI:** 10.1002/chem.201704845

**Published:** 2017-12-27

**Authors:** Christos Apostolidis, Michał S. Dutkiewicz, Attila Kovács, Olaf Walter

**Affiliations:** ^1^ European Commission–Joint Research Centre Directorate for Nuclear Safety and Security–G.I.5, Postfach 2340 76125 Karlsruhe Germany; ^2^ EaStCHEM School of Chemistry University of Edinburgh, The King's Buildings Edinburgh EH9 3FJ UK

**Keywords:** actinides, cyclopentadienyl ligands, metal-organic frameworks, plutonium, uranium

## Abstract

The organometallic tris‐cyclopentadienide actinide(III) (AnCp_3_) complexes were first reported about 50 years ago. However, up until now, only the NpCp_3_ solid state structure has been studied. Here we report on the solid state structures of UCp_3_ and PuCp_3_ which are isostructural to the Np analogue. The structural models are supported by theoretical calculations and compared to their lanthanide analogues. The observed trends in changes of bond lengths might be indicator for an increased covalency in the bonding in the tris‐cyclopentadienide actinide(III) complexes (AnCp_3_) compared to their lanthanide homologues.

The organometallic actinide chemistry with cyclopentadienyl ligands was developed in Karlsruhe and Munich by the pioneering work of E. O. Fischer, F. Baumgärtner, and B. Kanellakopulos together with P. Laubereau, then of the National Laboratories at Oak Ridge.

The oxidation state +III is not the most stable for all actinides. Nevertheless the solvent free non‐stabilized tris‐cyclopentadienide actinide(III) complexes AnCp_3_ were reported 50 years ago,^1^ a few years after the first reports on the AnCp_4_ complexes.^2^ Type LnCp_3_ (Ln: lanthanide) complexes not stabilized by Lewis base adduct formation have been previously studied.^3^ However, as the first example of an non‐stabilised AnCp_3_ complex, the synthesis and solid‐state structure of NpCp_3_ has only recently been published.^4^ This was followed by the first report on a structurally characterized organometallic Pu^III^ complex Pu(Cp(TMS)_2_)_3_ and its reduced Pu^II^ analogue^5^ and then the first report on a Pu^IV^ organometallic plutonocene derivative.^6^

Fifty years after the first reports, the structures of the UCp_3_ or PuCp_3_ complexes are still unknown. This is because even in the case of the adduct free LnCp_3_ complexes, high quality single crystals are not easily obtained. Indeed different forms are sometimes observed depending on the crystallization conditions.3c In case of the actinides, additionally, aging of solids is observed: after some weeks of storage they show drastically decreased solubility.1c This effect is however less noticeable when pure single crystalline material is stored.

Here, we close the knowledge gap on the solid‐state structures of AnCp_3_ (An: U, Pu). Comparing them to the structures of NpCp_3_ and related LnCp_3_ complexes offers the opportunity to gain a more detailed insight in the bonding. This is important for the understanding of 4f or 5f electron behaviour and differences therein.

UCp_3_ was prepared by reductive elimination of chloride from UCp_3_Cl with sodium amalgam in diethylether. PuCp_3_ was obtained from the direct reaction of PuCl_3_ with a slight excess of KCp. Both were purified by filtration and evaporation of the solvent followed by extraction with pentane or pentane/Et_2_O mixtures. The IR spectroscopic data reveal a fingerprint consistent with that previously reported for UCp_3_ and PuCp_3_.1b,1c The ^1^H NMR spectra of UCp_3_ show one single resonance at *δ*_H_=−15.60 ppm ([D_8_]THF) or −13.62 ppm ([D_3_]MeCN) for the formed adducts under these conditions, which are in agreement with literature‐known values.^7^ The cross‐peak for the CH C‐atom is observed at low field at 272.4 ppm in the ^13^C frequency resulting in an overall comparable situation as observed in the bis‐TMS substituted uranocene derivative in7b (Figure S1). The NMR spectroscopic investigations on PuCp_3_ are the 4^th^ example of a Pu organometallic complex for which a proton resonance is reported and the 2^nd^ complex on which multi‐dimensional NMR spectroscopy was performed.^5^, ^6^ In [D_6_]benzene there is one resonance observed for [PuCp_3_(thf)] at 11.59 ppm (in good agreement with the values reported in Ref. [5]) giving rise to a cross‐peak in the CH correlated spectrum at 81.4 ppm (Figure S2). This is a sign that the Cp rings are in equilibrium due to fast chemical exchange in the sample. It seems that in all Pu organometallic complexes reported up to now the chemical shifts observed for the proton as well as for the ^13^C resonances appear in the same range independently on the oxidation state of the metal being +II, +III, or +IV.^5^, ^6^, 7b

By extraction single crystals are obtained suitable for X‐ray diffraction analyses (Figure 1, all experimental details see the Supporting Information). Both compounds, UCp_3_ and PuCp_3_, form crystals that are isomorphic to the NpCp_3_ analogue.^4^ For Cm and Bk, the cell parameters have been identified by Debye–Scherrer analyses together with a series of LnCp_3_ complexes1e,1f all containing one axis doubled. Also discussed are some structures of LnCp_3_ complexes with comparable cell parameters, maybe containing one axis doubled but also with an identical reduced cell.^3^ Most of these structures show disorder of the Cp rings, and data collection was performed at room temperature. Both these factors prevent a good determination of the atom positions concerned, which leads to high standard deviations in distances and angles and makes any discussion on a significant level more difficult (see Baisch et al.3c). Therefore we performed our diffraction analyses at a temperature of 100 K in order to collect datasets of good quality. We describe the systems as orthorhombic Cmc2_1_ with *a*≈14.15, *b*≈8.70, and *c*≈9.60 Å, which corresponds to a monoclinic reduced cell of *a*≈8.30, *b*≈9.60, and *c*≈8.30 Å with *β* ≈116.5° (rounded values from all three data sets). The monoclinic cell has been used before to describe LaCp_3_ 3a and PrCp_3_ 3b whereas the orthorhombic cell was applied in the case for one PrCp_3_ structure which has been deposited at the CCDC3f but the space group reported is with Pbnm different from our findings.

**Figure 1:**
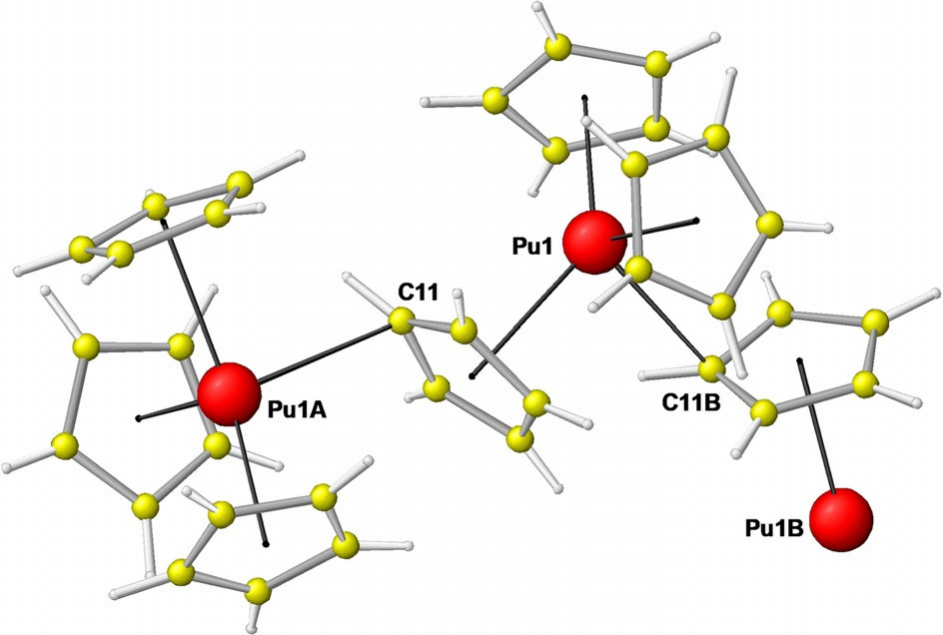
View of a part of the polymeric chain formed in the molecular structure of PuCp_3_ in the crystal, atoms indexed with A,B are symmetry generated. UCp_3_ forms isostructural crystals and shows identical molecular design.

We are now convinced that at least in the cases for the three actinide complexes AnCp_3_ (An: U, Np, Pu) the description in the orthorhombic space group Cmc2_1_ is best, as in the monoclinic reduced cell for the refinement a disorder must be introduced which is not the case in the orthorhombic cell. This leads for the monoclinic case in the refinement with identical crystallographic independent cell volume to nearly double the refined parameters but higher R values. As the two compounds UCp_3_ and PuCp_3_ form the same structure, only PuCp_3_ is depicted representatively in Figure 1.

In the sphere of the metal all Cp rings show η^5^‐coordination. The Lewis acidity of the actinides causes the formation of one additional η^1^‐coordination to one Cp ring of a neighboured AnCp_3_ residue; this Cp ring is μ‐η^5^,η^1^‐coordinated (bridging atom C11, Figure 1). This results in the polymeric zig‐zag structure motif which is known from the complexes LnCp_3_.^3^ We can exclude an interaction on the base of a μ‐η^5^,η^2^‐coordinated bridging cyclopentadienyl group as described earlier3b for the AnCp_3_ complexes also for all LnCp_3_ complexes whose solid state structures we have determined in the past years resulting in low temperature high quality datasets.^8^

A coordination environment of four Cp rings three establishing η^5^‐ and one η^1^‐coordination is also established in K[NpCp_4_] the KCp adduct to NpCp_3_.^4^ A symmetrical bonding of the η^5^‐ coordinated Cp rings is produced (mean Np‐Ct_Cp_ 251 pm, see footnote Table 1) together with a closer interaction to the η^1^‐ coordinated C‐atom of the fourth Cp ring (Np−C 275.2(7) pm) showing that Cp in KCp is a better Lewis base than in NpCp_3_. Lewis base adduct formation like in [UCp_3_(thf)] or in [UCp′_3_(quinuclidine)] produces a similar situation with symmetrical η^5^‐coordination of the Cp rings with a closer interaction to the donor atom of the Lewis base involved than observed here for the μ‐η^1^‐coordinated C‐atom.^9^

**Table 1:** Selected bond lengths [pm].

	UCp_3_	NpCp_3_ 4	PuCp_3_
M−C(μ‐η^1^) ^[a]^	278.1(23) 293.7(23)	281.4(15) 289.4(15)	283.0(12) 288.8(12)
M−Ct_Cp_^[b]^	241.6	241.9	239.2
M−Ct_Cp_^[c]^	260.4/260.8	256.1/258.7	256.5/257.4
M−C^[b]^	265.8–274.7; 270.1 ^[d]^	266.8–273.6; 270.3 ^[d]^	264.4–272.0; 267.9 ^[d]^
M−C^[c]^	279.4–293.7; 287.2 ^[d]^	278.9–292.2; 284.3 ^[d]^	276.9–291.5; 283.9 ^[d]^

Standard deviations in parentheses only for dedicated bonds not for calculated ideal positions or ranges. Ct_Cp_: idealised position of center of Cp ring. [a] First value for η^1^‐, 2^nd^ value for η^5^‐coordination. [b] Cp ring closer to the An. [c] Cp rings more distant to the An. [d] mean value.

The bonding of the three Cp rings in η^5^‐coordination in AnCp_3_ (An: U, Np, Pu) is not symmetrical: one of the rings (not the one involved in the bridging mode) in all the three structures, is localized closer to the central An^III^ ion than the other two (Table 1). This is also the case for the recently studied complex Pu(CpTMS_2_)_3_.^5^ This behaviour supports the high coordinative flexibility of both the Cp rings and the actinide ions.

In agreement with the asymmetrical bonding of the Cp rings the U−C bond lengths for the Cp ring closer to the coordinated metal are 265.8 to 274.7 pm, for the other two Cp rings 279.4 to 293.7 pm. The corresponding values for the PuCp_3_ are 264.4 to 272.0 and 276.9 to 291.5 pm, respectively. Accordingly the distances between metal ions and the centres of the Cp rings (Ct_Cp_ in Table 1) are found to 241.6, 260.4, 260.8 pm (U) and 239.2, 256.5, 257.4 pm for PuCp_3_. For the series U, Np, Pu one can see, that the Cp rings approach to the metal about 3 pm (Table 1). This is reflected as well in the mean An−C bond lengths (Table 1). The effect is comparable to the one observed for the lanthanide complexes LnCp_3_ [see Figure S3, right] and might be attributed to actinide contraction. As the η^5^‐π‐coordinated Cp ring approaches the An^III^ ion centres the η^1^‐interaction to the μ‐η^5^,η^1^‐coordinated C atom decreases. This results in an elongation of the bond length M‐C(μ‐η^1^) from 278(2) for UCp_3_ over 281(2) for NpCp_3_ to 283(1) pm for PuCp_3_ (Table 1). This increase of ≈5 pm describes a trend; the high standard deviations disable to make a clear statement based only on experimental data. However, over the series of the three complexes the elongation of the η^1^‐interaction to the μ‐η^5^,η^1^‐coordinated C atom of ≈5 pm seems to be about twice as much as that observed for the corresponding lanthanide complexes [see Figure S3, left]. So in the case of the complexes MCp_3_, this bond might possibly be regarded as an indicator for changes in the metal electronic environment.

This is because the outer orbitals of the actinide ions in AnCp_3_ reach out far enough to establish a good interaction to the π‐coordinated Cp rings at the given distance demonstrating again the high coordinative flexibility of both the Cp rings and the actinide ions. This hypothesis is supported by the results from DFT calculations we performed using a dimeric molecular model of selected Ln and An complexes reducing the structural motif to a negatively charged unit (Cp_3_‐M‐Cp‐M‐Cp_3_)^−^ with the central Cp ring in the bridging position (details see Supporting Information and Figure S3). The geometry optimisations reproduced the η^5^,η^1^‐coordination of the bridging Cp ring, confirming that this unique interaction belongs to the basic bonding properties of the complexes and is not enforced by the packing effects. Similarly, the competitive nature of η^5^,η^1^‐interactions are confirmed by the calculations, the results reflecting the already described changes in the M−C distances. During the geometry optimisations we observed that the system is very flexible; it exhibits a flat potential energy hyperface. Hence slight changes in force can cause significant changes in the structure in the η^1^‐M−C distances. Another significant clue on the bonding was the verified importance of the 4f subshell for the Ln−Cp donor–acceptor interactions, calculations using the 4f‐in‐core Ln pseudopotentials failed to reproduce the characteristic change of the η^1^‐Ln−Cp distances. On the other hand, the experimentally suggested gradual change in the M−C bond lengths for η^5^‐ and η^1^‐coordinated Cp rings across the 4f/5f rows were only partially reproduced by the calculations. The probable reason lies in the already mentioned flat potential energy surface and the dimeric model structure (size limited by technical problems in the calculations) being unable to account for long‐range cooperative or solid‐state effects.

Our experimental results described here close the knowledge gap on the solid state structure of the long known complexes PuCp_3_ and UCp_3_. They indicate that covalency in AnCp_3_ is higher than in LnCp_3_ (at least for the here reported minor actinide complexes), which is in agreement with theoretical considerations.^10^ Series comparing experimental data of transition metal or lanthanide complexes to their actinide analogues together with theoretical calculation showed in other cases as well: 5f and or 6d orbital contribution contributes to covalency in the bonding of actinide complexes. It is influenced by the interplay between the metal ions and the ligands.^11^

With this background it seems promising to compare as well the cyclohexylisonitrile adducts AnCp_3_(CNC_6_H_11_) to those of the corresponding lanthanides. The IR CN‐stretching vibration of the isonitrile ligand is an excellent sensor on its binding mode and forces which enables the detection of differences between the lanthanides and actinides in their complexes MCp_3_(CNC_6_H_11_).1c, 12

## Crystallographic data

CCDC 570389 (PuCp_3_) and 1570390 (UCp_3_), contain the supplementary crystallographic data for this paper. These data are provided free of charge by The Cambridge Crystallographic Data Centre. For further information, please see the Supporting Information.

## Conflict of interest

The authors declare no conflict of interest.

## Supporting information

As a service to our authors and readers, this journal provides supporting information supplied by the authors. Such materials are peer reviewed and may be re‐organized for online delivery, but are not copy‐edited or typeset. Technical support issues arising from supporting information (other than missing files) should be addressed to the authors.

Supplementary
