# Supplementary material for: Solid‐State Structure of Tris‐Cyclopentadienide Uranium(III) and Plutonium(III)
Source: Chemistry. 2017 Dec 27;24(12):2841–4. doi: 10.1002/chem.201704845 (PMC5861669; doi:10.1002/chem.201704845)
Supplement: Supplementary file 1 — Supplementary [file CHEM-24-2841-s001.pdf]

CHEMISTRY
A European Journal

Supporting Information

Solid-State Structure of Tris-Cyclopentadienide Uranium(III) and Plutonium(III)

Christos Apostolidis,[a] Michał S. Dutkiewicz,[a, b] Attila Kovács,[a] and Olaf Walter*[a]

chem_201704845_sm_miscellaneous_information.pdf

Table of Contents

Experimental procedures	page 2
NMR spectra	page 3
Xray analyses	page 3
DFT calculations	page 4
Author contributions	page 6
References	page 6
Appendix, Cartesian coordinates of calculated structures	page 7

Experimental Procedures

The manipulations with radionuclides were conducted in the radiochemical laboratories at the Joint Research Centre (JRC) – Karlsruhe, Germany. Unsealed transuranium compounds were manipulated in dinitrogen filled (99+%), negative-pressure radiological gloveboxes. The glovebox for preparative chemistry was fitted with an automated dual vacuum/argon manifold and standard Schlenk techniques were used.
$PuCp_3$ can be prepared as described in [1] or according to the following procedure.
150 mg of $PuCl_3$ (0.43 mmol) were added to a suspension of 154 mg (1.48 mmol) KCp in 50 ml of THF. The mixture was heated to reflux for 7 d. The solvent was removed in vacuum. 1 ml of toluene is added and removed in vacuum, the residue was dried in UHV for 8h at RT and then extracted with pentane yielding in dark green crystals of $PuCp_3$ (123 mg, 0.28 mmol, 66%).
UCp_3 can be prepared according to [2] or according to the following procedure.
A 50-mL Schlenk tube was charged with UCp_3Cl, (1.3667 g, 2.916 mmol), solid sodium amalgam (1.399 g, 5.03 %, 1.05 eq) and mercury metal (12.677 g). Diethyl ether (35 mL) was added and the reaction mixture was stirred for 24 h. During this time a dark brown/grey suspension formed and the dark brown supernatant isolated by filtration (fritted glass disk, 10-16 µm porosity). The filter cake was extracted with diethyl ether until no visible change in fresh eluent colour and the combined extracts evaporated to dryness under vacuum (40 °C, 3×10^{-3} mbar, 4 h) (i). The resulting solid was extracted with 6 $\%_{v/v}$ diethyl ether in *n*-pentane for 10 d. The resulting dark brown, almost black single crystals of UCp_3 were collected and dried under vacuum (20 °C, 6×10^{-4} mbar, 6 h, yield 569 mg, 45 %).
The ^1H NMR spectra were recorded on the Bruker Ascent™ 400 MHz WB NMR/DNP spectrometer equipped with an inverse Z-gradient Bruker Triple Resonance Broad Band Probe (TBI). Degassed fluoropolymer NMR tube liners (4 mm nominal O.D.; 140°C, 6×10^{-4} mbar, 12 h) were charged with the liquid samples ensuring that the outer surface remained free from contamination, and sealed. The sealed liner was then transferred into a standard borosilicate glass NMR tube of which the upper part was placed in PVC bag. The bag which was connected to the sample glovebox was welded then. The sample was measured normally. Chemical shifts were calibrated against residual proton solvent signal and are reported relative to tetramethylsilane (δ = 0 ppm).
^1H NMR data of UCp_3 have been reported earlier in the literature [3], our data are in agreement. For $PuCp_3$ no NMR data are reported up to now, but as the paramagnetism of $PuCp_3$ is not really high NMR spectra can be recorded even enabling to register a CH correlated spectrum (Figure S1). Two cross peaks can be identified, the one at 11.59 (1H) / 81.4 (13C) ppm arises from the $PuCp_3$.
IR data were registered on a Bruker Alpha II IR spectrometer placed in radiation protection glovebox. Data were absolutely consistent with the ones already reported [1b].

WILEY-VCH

NMR spectra

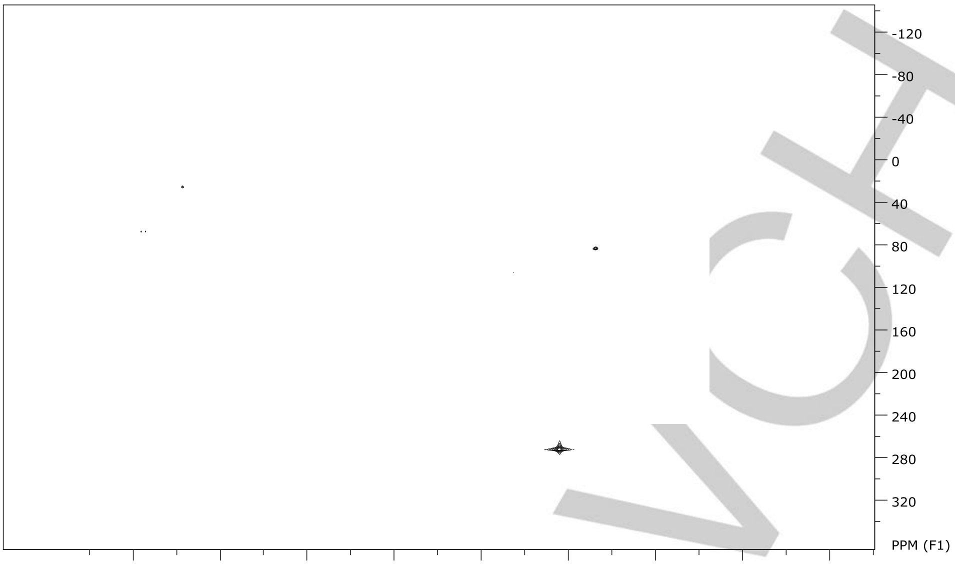

Figure/Scheme S1. CH correlated NMR spectrum of UCp_3 in d^8-thf. The cross peaks at -15.60 (^1H) / 272.4 (^{13}C) ppm is assigned to the CH of the Cp rings in UCp_3, the signals at 3.75 and 1.73 ppm (^1H) arise from the thf whereas the other resonances at -13.4 / 105.5 and -17.1 / 83.1 correspond to impurities containing UCp units.

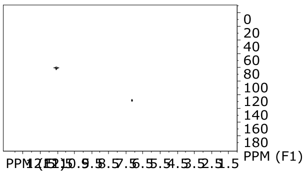

Figure/Scheme S2. CH correlated NMR spectrum of $PuCp_3$ in d^6-benzene. Two cross peaks can be identified the one at 11.59 (^1H) / 81.4 (^{13}C) ppm arises from the $PuCp_3$, the 2nd is allocated to C_6HD_5.

X-ray analyses

XRD measurements were performed on a Bruker Apex II Quazar diffractometer with monochromated MoKα-radiation collecting four full spheres of data [4] built by 2844 frames. A combined ω– and φ-scan technique was employed for data collection with $\Delta\omega = \Delta\varphi = 0.5°$ and irradiation times of 2 s ($PuCp_3$) or 4 s (UCp_3) per frame appropriate to size and diffracting abilities of the crystals. Data were integrated with SAINT [SAINT-Plus], corrected to Lorentz and polarisation effects and an adsorption correction with SADABS [4] was applied. The structures were solved by direct methods and refined to an optimum R_1 value with shelx-2013 [5]. Visualisation for evaluation was performed with xpma [6] and figure were created with winray-32 [7].

The structures have been deposited at The Cambridge Crystallographic Data Centre with the reference CCDC numbers 1570389 ($PuCp_3$) and 1570390 (UCp_3), they contain the supplementary crystallographic data for this paper. These data can be obtained free of charge from the CCDC via www.ccdc.cam.ac.uk/data_request/cif.
The compounds crystallise in a chiral space group, but they show racemic twinning, which is confirmed by the Flack x parameter close to 0.5. Due to the symmetry of the cell in crystallographic independent part of the elementary cell only half a molecule is found. The generation of the other half of the molecule corresponds to a disorder of the Cp rings. This is one reason why the standard deviations in the An-C bond lengths are found to be relatively high. Another one is systematic for all actinide compounds: C atoms in the neighbourhood of an actinide atom do not give a high contrast in x-ray diffraction; the situation is comparable to an H atom in the neighbourhood of a P atom. This situation is becoming even worse when the heavy metal atom is located on a special position. So the relatively high standard deviations in the bond lengths are quite normal. The high electron number of the actinides is as well the reason for the higher than usual residual electron density which is quite common in actinide compounds.
As the data quality for $PuCp_3$ is better the atomic positions in the cell are described better and the standard deviations are smaller.

Bond lengths for UCp_3 (Å):
U(1)-C(1) 2.84, U(1)-C(2) 2.90, U(1)-C 2.92, U(1)-C(4) 2.88, U(1)-C(5) 2.83, U(1)-C(6) 2.75(2), U(1)-C(7) 2.71, U(1)-C(8) 2.66, U(1)-C(9) 2.67, U(1)-C(10) 2.72, U(1)-C(11) 2.94, U(1)-C(12) 2.93, U(1)-C(13) 2.84, U(1)-C(14) 2.79, U(1)-C(15) 2.85, U(1)-C(11A) 2.78(4), U(1)-C(12A) 3.56, U(1)-C(13A) 4.27, U(1)-C(14A) 4.09, U(1)-C(15A) 3.20. Standard deviation is 0.03 Å.

Bond lengths for $PuCp_3$ (Å):
PU(1)-C(1) 2.80, Pu(1)-C(2) 2.86, Pu(1)-C(3) 2.88, Pu(1)-C(4) 2.84, Pu(1)-C(5) 2.79, Pu(1)-C(6) 2.72, Pu(1)-C(7) 2.69, Pu(1)-C(8) 2.64, Pu(1)-C(9) 2.65, Pu(1)-C(10) 2.70, Pu(1)-C(11) 2.89, Pu(1)-C(12) 2.91, Pu(1)-C(13) 2.84, Pu(1)-C(14) 2.77, Pu(1)-C(15) 2.80, Pu(1)-C(15) 2.85, Pu(1)-C(11A) 2.83, Pu(1)-C(12A) 3.59, Pu(1)-C(13A) 4.32, Pu(1)-C(14A) 4.17, Pu(1)-C(15A) 3.28. Standard deviation is 0.02 Å.

Table S1. Crystallographic details.

	UCp_3	$PuCp_3$
formula	C_{15} H_{15} U	C_{15} H_{15} Pu
Formula weight	433.30	437.27
Temperature	100(2) K	100(2) K
Wavelength	0.71073 Å	0.71073 Å
Crystal system	Orthorhombic	
Space group	$Cmc2_1$	
Unit cell dimensions	a = 14.191(2) Å	a = 14.099(3) Å
	b = 8.7611(13) Å	b = 8.6930(17) Å
	c = 9.5889(14) Å	c = 9.6144(19) Å
Volume	1192.2(3) Å3	1178.4(4) Å3
Z	4	4
Density (calculated)	2.414 Mg/m^3	2.465 Mg/m^3
Absorption coefficient	13.583 mm^{-1}	5.561 mm^{-1}
F(000)	788	796
Crystal size	0.012 x 0.024 x 0.077 mm^3	0.024 x 0.044 x 0.083 mm^3
Theta range for data collection	2.732 to 28.563°	2.753 to 28.445°
Index ranges	-18<=h<=18, -11<=k<=11, -12<=l<=12	-17<=h<=18, -11<=k<=11, -12<=l<=12
Reflections collected	9639	10359
Independent reflections	1509 [R(int) = 0.0442]	1470 [R(int) = 0.0206]
Completeness to θ = 25.000°	99.8 %	100.0 %
Refinement method	Full-matrix least-squares on F^2	
Data / restraints / parameters	1509 / 91 / 116	1470 / 91 / 116
Goodness-of-fit on F^2	1.513	1.346
Final R indices [I > 2 σ]	R_1 = 0.0423, wR_2 = 0.0946	R_1 = 0.0211, wR_2 = 0.0498
R indices (all data)	R_1 = 0.0438, wR_2 = 0.0956	R_1 = 0.0214, wR_2 = 0.0499
Absolute structure parameter	0.49(8)	0.50(10)
Largest diff. peak and hole	4.730 and -5.393 eÅ-3	2.343 and -2.454 $eÅ^{-3}$

Standard deviations in parentheses.

Trends in bond lengths by comparison to the lanthanide analogues (Figure S3):

- $AnCp_3$ shows a closer η^1-M-C distance for the μ-η^5,η^1-coordinated C atom in the complexes MCp_3 than the lanthanides
- the η^1-M-C distance for the μ-η^5,η^1-coordinated C atom in the complexes MCp_3 increases with increasing atomic number
- the increase of η^1-M-C distance for the μ-η^5,η^1-coordinated C atom in the complexes MCp_3 is stronger for An than for Ln
- the metal to centre of Cp ring distance in the complexes MCp_3 (M: Ln, An) for the η^5-coordinated Cp rings decreases with increasing atomic number
- this decrease is parallel for Ln and An

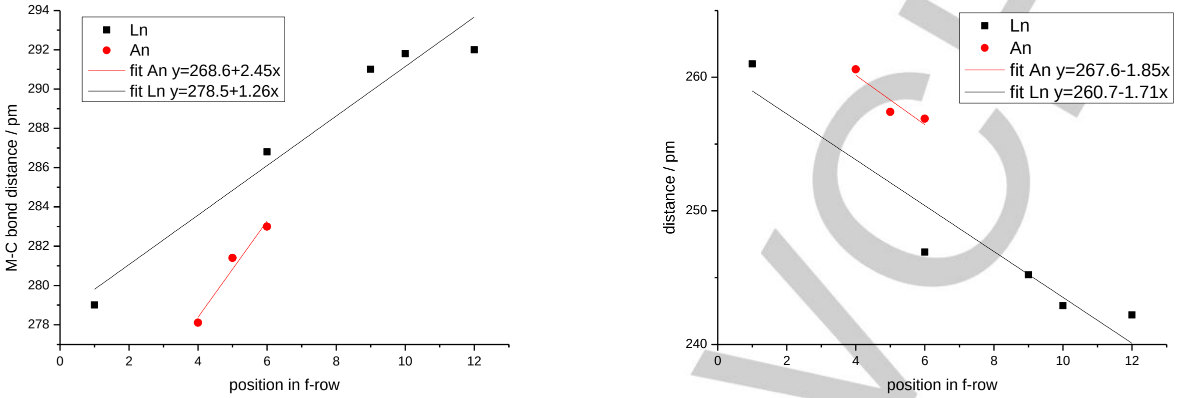

Figure/Scheme S3. Left: η^1-M-C distance for the μ-η^5,η^1-coordinated C atom in the complexes MCp_3 (M: Ln, An).Right: Metal to centre of Cp ring distance in the solid state structures of the complexes MCp_3 (M: Ln, An). Error bars neglected for reasons of clarity.

DFT calculations, computational details

The $[Cp_2\text{-}M\text{-}Cp\text{-}]_n$ chain was approximated by a $(Cp_3\text{-}M\text{-}Cp\text{-}M\text{-}Cp_3)^-$ model structure containing two Ln or An atoms. Accordingly, the model has one negative charge. Calculations on a larger model containing four metals (Sm, Dy) failed because of SCF convergence problems occurring very often for such complex open-shell systems. The Cartesian coordinates of the computed (optimized) structures are given below in the Supporting Information.

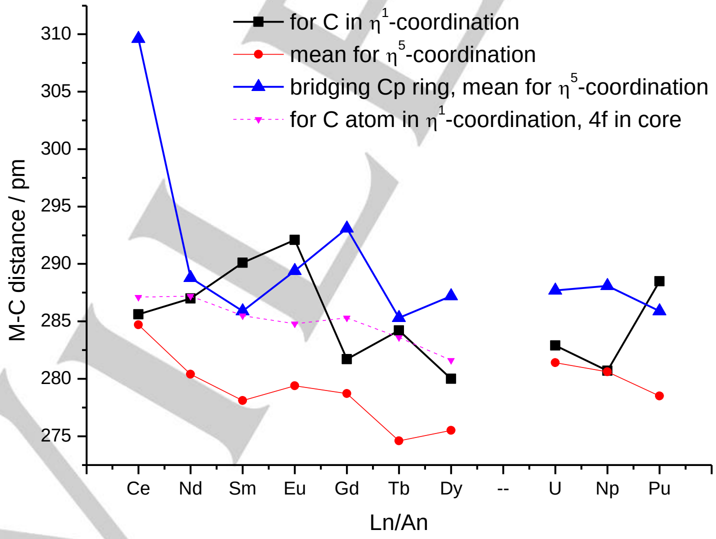

Figure/Scheme S4. Distance in the structures of the calculated structures.

The computations were performed with the Gaussin09 suit of the Gaussian programs [8] using the B3LYP [9] exchange-correlation functional. Both 4f-in-core and small-core 4f-in-valence quasi-relativistic pseudopotentials of the lanthanides were applied in order to assess the importance of the 4f subshell in the bonding [10]. The valence basis set for the former pseudopotential treating the 5s5p5d6s orbitals had the contraction scheme of [8s7p6d3f2g]/[6s5p5d3f2g] [11]. The valence basis set for the latter small-core pseudopotentials treating the 4s4p4d4f5s5p5d6s orbitals had the contraction scheme of [14s13p10d8f6g]/[10s8p5d4f3g] [12]. For C and H the standard 6-31G** basis set was used. Test calculations with the 6-311+G** basis set resulted uniformly (by 1 pm) larger

WILEY-VCH

Ln-C distances for the η^1-coordinated C atom in the Cp ring, hence the trend is preserved with the cost-effective double-zeta basis set. For the actinides U, Np and Pu the small-core 5f-in-core quasi-relativistic pseudopotentials (ECP60MWB) [13] were applied in conjunction with a 14s13p10d8f6g valence basis set contracted to 10s9p5d4f3g [13b].
The B3LYP functional was extended with the D3 version of Grimme's dispersion correction using the original D3 damping function (D3) [14]. The importance of dispersion effects was shown by test calculations: without the D3 corrections the Ln-C distances for the η^1-coordinated C atom in the Cp ring for Ln = Sm / Dy were practically identical, while the B3LYP-D3 calculations using the 4f-in-core Ln pseudopotentials resulted in a significant difference of 4 pm. Being important for weak interactions, the SuperFine grid was applied for integration accuracy. It contains 150 radial shells and 974 angular points per shell for C and H, while 225 radial shells for the lanthanides and actinides.
The spin multiplicities of the model structures with 4f/5f-in-core basis sets corresponded to the electronic structures of the M^{3+} ions: for the pairs of Ce, Nd, Sm, Eu, Gd, Tb, Dy, U, Np and Pu they were 3, 7, 11, 13, 15, 13, 11, 7, 9 and 11, respectively. Test calculations on the Nd complex with spin multiplicities 5 and 3 as well as spin multiplicity 1 on the Ce complex revealed the insensitivity of the Ln-C interaction for the η^1-coordinated C atom in the Cp ring (change of 0.1 pm) on the electronic structure of the Ln ion. In contrast, the spin multiplicity 5 of the U complex showed a decrease of 1.4 pm, while the spin multiplicity 7 of the Np complex a decrease of 0.3 pm. The higher energies from the latter test calculations confirmed also the ground-state character of the above given spin multiplicities. The lowest-energy electronic structure of the open-shell complexes was managed by employing the Stable keyword of Gaussian09. In addition, for the Np complex we probed several manual alterations of the 5f orbital populations, but all these altered populations changed back to the original one.

Main conclusions from the calculations (Figure S3):
1. Importance of dispersion forces in the metal-ligand interactions (application of dispersion correction in the DFT calculations).
2. Importance of 4f subshell for the donor-acceptor interaction (though the valence basis sets of the 4f-in-core pseudopotentials include f and g polarisation functions, there are not sufficient for the description of the metal-ligand interaction in these complexes).
3. Unimportance of the electronic structure of the lanthanides for the metal-ligand interaction; in contrast, some significance of the electronic structure of the actinides.
4. Flat potential energy surface in the M-Cp-M moiety (shown by oscillation of the M-C distances for the η^1-coordinated C atom of the Cp ring up to 2 pm in the final steps of the geometry optimisations).
5. The changes in the M-C distances for the η^1-coordinated C atom in the Cp ring correlate well with the changes of the η^5-coordinated Cp ring M-C distances to the opposite direction (Figure S3).
6. The computations failed to reproduce a uniform increase of the M-C distances for the η^1-coordinated C atom in the Cp ring along the Ln/An rows (cf. Figure). A possible reason is the above mentioned flat potential energy surface around the M-Cp-M moiety and the necessarily small size of the model structures. The latter restriction cannot account for long-range cooperative or solid-state effects.

Author Contributions

Christos Apostolidis and Michał S. Dutkiewicz have performed the syntheses, spectroscopic verification of the compounds, and crystal growth. Attila Kovacs performed the theoretical calculations. Olaf Walter performed the single crystal x-ray diffraction analyses and NMR spectroscopic investigations.

References

[1] F. Baumgärtner, E. O. Fischer, B. Kanellakopulos, P. Laubereau, *Angew. Chem.* **1965**, *77*, 866; *Angew. Chem. Int. Ed.* **1965**, *4*, 878.
[2] B. Kanellakopulos, E. O. Fischer, E. Dornberger, F. Baumgärtner, *J. Organomet. Chem.* **1970**, *24*, 507-514.
[3] G. Folcher, H. Marquet-Ellis, P. Rigny, E. Soulié, G. Goodman, *Journal of Inorganic and Nuclear Chemistry* **1976**, *38*, 747-753.
[4] Bruker , APEX2, SAINT-Plus, SADABS, Programs for data collection, integration and absorption correction, Bruker AXS Inc., Madison, Wisconsin, USA, **2007**.
[5] a) G. M. Sheldrick, Acta *Cryst.* **2008**, *A64*, 112-122. b) G. M. Sheldrick, *Acta Cryst*. **2015**, *C71*, 3-8.
[6] L. Zsolnai, G. Huttner, xpma, University of Heidelberg, **1994**.
[7] R. Soltek, G. Huttner, winray-32, University of Heidelberg, **1998**.
[8] M. J. Frisch, G. W. Trucks, H. B. Schlegel, G. E. Scuseria, M. A. Robb, J. R. Cheeseman, G. Scalmani, V. Barone, B. Mennucci, G. A. Petersson, H. Nakatsuji, M. Caricato, X. Li, H. P. Hratchian, A. F. Izmaylov, J. Bloino, G. Zheng, J. L. Sonnenberg, M. Hada, M. Ehara, K. Toyota, R. Fukuda, J. Hasegawa, M. Ishida, T. Nakajima, Y. Honda, O. Kitao, H. Nakai, T. Vreven, J. A. Montgomery Jr., J. E. Peralta, F. Ogliaro, M. Bearpark, J. J. Heyd, E. Brothers, K. N. Kudin, V. N. Staroverov, T. Keith, R. Kobayashi, J. Normand, K. Raghavachari, A. Rendell, J. C. Burant, S. S. Iyengar, J. Tomasi, M. Cossi, N. Rega, J. M. Millam, M. Klene, J. E. Knox, J. B. Cross, V. Bakken, C. Adamo, J. Jaramillo, R. Gomperts, R. E. Stratmann, O. Yazyev, A. J. Austin, R. Cammi, C. Pomelli, J. W. Ochterski, R. L. Martin, K. Morokuma, V. G. Zakrzewski, G. A. Voth, P. Salvador, J. J. Dannenberg, S. Dapprich, A. D. Daniels, O. Farkas, J. B. Foresman, J. V. Ortiz, J. Cioslowski, D. J. Fox, Gaussian, Inc., Wallingford CT, **2010**.
[9] a) A. D. Becke, *J. Chem. Phys.* **1993**, *98*, 5648-5652. b) C. Lee, W. Yang, R. G. Parr, *Phys. Rev. B* **1988**, *37*, 785-789.
[10] a) M. Dolg, H. Stoll, H. Preuss, *J. Chem. Phys.* **1989**, *90*, 1730-1734. b) M. Dolg, H. Stoll, H. Preuss, *Theor. Chim. Acta* **1993**, *85*, 441-450. c) M. Dolg, H. Stoll, A. Savin, H. Preuss, *Theor. Chim. Acta* **1989**, *75*, 173-194.
[11] a) J. Yang, M. Dolg, *Theor. Chem. Acc.* **2005**, *113*, 212-224. b) A. Weigand, X. Cao, J. Yang, M. Dolg, *Theor. Chem. Acc.* **2009**, *126*, 117-127.
[12] X. Cao, M. Dolg, *J. Mol. Struct. (Theochem)* **2002**, *581*, 139-147.
[13] a) W. Küchle, M. Dolg, H. Stoll, H. Preuss, *J. Chem. Phys.* **1994**, *100*, 7535-7542. b) X. Cao, M. Dolg, H. Stoll, *J. Chem. Phys.* **2003**, *118*, 487-496.
[14] S. Grimme, J. Antony, S. Ehrlich, H. Krieg, *J. Chem. Phys.* **2010**, *132*, 154104.

Appendix, cartesian coordinates of calculated structures

Ce				Nd				Sm				Eu				Gd			
C	4.334754	-1.9757	-1.58696	C	4.236118	-2.56594	0.56639	C	4.212712	-2.55758	0.590047	C	4.294661	-2.52998	0.619601	C	4.228035	-2.42086	0.570953
H	5.092023	-1.57927	-2.2505	H	4.949153	-2.91257	-0.16939	H	4.922624	-2.92536	-0.13831	H	5.006781	-2.89483	-0.10802	H	4.983059	-2.66835	-0.16106
C	2.962654	-2.1323	-1.88954	C	2.842814	-2.80183	0.550312	C	2.811643	-2.76843	0.568029	C	2.891497	-2.77464	0.609145	C	2.83258	-2.69577	0.458278
H	2.478129	-1.86904	-2.82113	H	2.295242	-3.35362	-0.2026	H	2.2589	-3.31675	-0.18316	H	2.348983	-3.34703	-0.13155	H	2.342745	-3.1736	-0.37904
C	2.325463	-2.68389	-0.74662	C	2.281927	-2.17233	1.692767	C	2.257474	-2.12321	1.701488	C	2.33072	-2.13038	1.733501	C	2.209529	-2.24051	1.64707
H	1.276572	-2.93635	-0.66441	H	1.23746	-2.1837	1.974997	H	1.21181	-2.11111	1.977206	H	1.286247	-2.1341	2.01348	H	1.153376	-2.30328	1.870768
C	3.300795	-2.86005	0.258021	C	3.326342	-1.54685	2.405973	C	3.309736	-1.5075	2.413338	C	3.374793	-1.47562	2.42832	C	3.203667	-1.69198	2.484227
H	3.126093	-3.2508	1.251332	H	3.220081	-0.977	3.318701	H	3.209277	-0.93046	3.322027	H	3.268085	-0.89586	3.334759	H	3.035058	-1.22766	3.447143
C	4.543673	-2.40775	-0.25258	C	4.535442	-1.77194	1.701228	C	4.520476	-1.7634	1.719575	C	4.590684	-1.71933	1.733341	C	4.446471	-1.78914	1.817925
H	5.490539	-2.41711	0.273534	H	5.518319	-1.42332	1.993939	H	5.507225	-1.42846	2.015037	H	5.570039	-1.35573	2.017914	H	5.401579	-1.44208	2.191243
C	3.912791	1.166455	-2.45843	C	3.751291	-1.38002	-2.39768	C	3.722574	-1.41351	-2.3657	C	3.76955	-1.42637	-2.34906	C	3.829284	-1.33135	-2.47697
H	3.992515	0.473356	-3.28543	H	3.742368	-2.46035	-2.34818	H	3.716414	-2.49329	-2.3079	H	3.784391	-2.50577	-2.28317	H	3.855816	-2.41302	-2.49796
C	2.757172	1.889053	-2.09363	C	2.647636	-0.55916	-2.70973	C	2.611264	-0.5973	-2.66638	C	2.642995	-0.63373	-2.67045	C	2.698308	-0.5305	-2.7324
H	1.788574	1.837656	-2.57198	H	1.64199	-0.89196	-2.92627	H	1.601532	-0.93375	-2.85598	H	1.638514	-0.9904	-2.85126	H	1.70599	-0.87854	-2.98129
C	3.069354	2.664906	-0.94606	C	3.075829	0.7927	-2.64827	C	3.038416	0.752703	-2.62521	C	3.046393	0.718489	-2.64918	C	3.067539	0.826134	-2.53836
H	2.381665	3.315945	-0.42254	H	2.452433	1.658467	-2.82831	H	2.411424	1.616088	-2.80197	H	2.406938	1.569499	-2.84074	H	2.419621	1.682483	-2.66205
C	4.41858	2.418688	-0.6054	C	4.444663	0.802167	-2.29698	C	4.418247	0.767435	-2.30239	C	4.43283	0.761949	-2.32272	C	4.438114	0.853369	-2.15684
H	4.955471	2.856155	0.226143	H	5.062386	1.680715	-2.16671	H	5.036829	1.648532	-2.19551	H	5.034489	1.65654	-2.23334	H	5.019548	1.739336	-1.94559
C	4.938912	1.474787	-1.52726	C	4.861557	-0.54118	-2.11985	C	4.840568	-0.5714	-2.12383	C	4.877029	-0.56066	-2.12486	C	4.90077	-0.48338	-2.1048
H	5.948915	1.081797	-1.5365	H	5.857917	-0.86932	-1.84856	H	5.842291	-0.89709	-1.8703	H	5.88252	-0.86638	-1.86313	H	5.899161	-0.79756	-1.82677
C	2.654273	-0.6797	2.741384	C	2.81414	1.716845	2.160171	C	2.776111	1.719971	2.114896	C	2.816679	1.756614	2.110444	C	2.614022	1.57034	2.123933
H	2.103768	-1.59458	2.919698	H	2.258533	1.341913	3.009973	H	2.214021	1.351141	2.962821	H	2.279051	1.396561	2.977862	H	2.050234	1.086993	2.910155
C	2.095292	0.618545	2.634286	C	2.270461	2.424753	1.059665	C	2.240358	2.408959	1.001029	C	2.250322	2.425294	1.003101	C	2.087257	2.394305	1.100302
H	1.043815	0.861058	2.717608	H	1.228438	2.687975	0.933332	H	1.19716	2.655154	0.854819	H	1.201438	2.655601	0.873358	H	1.040046	2.627258	0.960323
C	3.14613	1.533786	2.401989	C	3.322872	2.732304	0.169494	C	3.301151	2.714031	0.120428	C	3.290222	2.726002	0.094975	C	3.158244	2.844267	0.297929
H	3.040771	2.600999	2.256788	H	3.227713	3.2541	-0.77326	H	3.213446	3.22387	-0.82931	H	3.179143	3.229502	-0.85591	H	3.072359	3.455588	-0.591

C	4.361098	0.80223	2.365914	C	4.523342	2.213005	0.718361	C	4.502806	2.219831	0.69455	C	4.513683	2.25603	0.654276	C	4.355909	2.302583	0.820705
H	5.349721	1.217164	2.211827	H	5.510161	2.290646	0.279426	H	5.493935	2.30412	0.266746	H	5.493462	2.344303	0.202622	H	5.351609	2.450863	0.423379
C	4.056356	-0.56374	2.584962	C	4.208269	1.594904	1.952875	C	4.178178	1.613508	1.929543	C	4.222343	1.663274	1.896951	C	4.02766	1.519093	1.952991
H	4.769135	-1.37737	2.617581	H	4.908903	1.104654	2.615716	H	4.874379	1.141604	2.610078	H	4.935733	1.207631	2.570633	H	4.722926	0.988478	2.587473
C	-3.63293	0.900521	2.639789	C	-3.52366	2.829847	0.797103	C	-3.5021	2.832818	0.763683	C	-3.47297	2.863678	0.767416	C	-3.46611	2.800376	0.804967
H	-3.78022	0.100473	3.350882	H	-3.57705	2.98811	1.865736	H	-3.5539	3.009906	1.829361	H	-3.51461	3.052988	1.831534	H	-3.50326	2.939603	1.876308
C	-4.60981	1.426333	1.762692	C	-4.54234	2.28149	-0.01492	C	-4.52077	2.258228	-0.03511	C	-4.5178	2.329918	-0.02666	C	-4.49044	2.24956	0.001437
H	-5.63879	1.098623	1.680309	H	-5.50818	1.927332	0.320479	H	-5.47997	1.898488	0.311974	H	-5.48853	2.007274	0.326255	H	-5.44797	1.884892	0.348377
C	-4.00371	2.479813	1.025428	C	-4.06097	2.240536	-1.34966	C	-4.04659	2.198596	-1.36896	C	-4.04763	2.243847	-1.35741	C	-4.02324	2.224677	-1.34071
H	-4.48251	3.097759	0.278397	H	-4.60189	1.863069	-2.20747	H	-4.58555	1.796579	-2.21639	H	-4.60307	1.868103	-2.20675	H	-4.56935	1.850133	-2.19652
C	-2.66078	2.582264	1.432086	C	-2.74797	2.756765	-1.35661	C	-2.73694	2.725957	-1.3905	C	-2.71524	2.729367	-1.38562	C	-2.71692	2.754251	-1.35914
H	-1.93807	3.285028	1.041969	H	-2.10574	2.849333	-2.22202	H	-2.09986	2.809558	-2.26047	H	-2.08011	2.786051	-2.25935	H	-2.08099	2.845051	-2.22891
C	-2.42006	1.593103	2.41995	C	-2.40492	3.105906	-0.02565	C	-2.39179	3.107288	-0.06813	C	-2.35562	3.101614	-0.07066	C	-2.3602	3.092608	-0.03011
H	-1.48238	1.412598	2.931027	H	-1.46534	3.535434	0.30083	H	-1.45432	3.550954	0.24513	H	-1.40251	3.510739	0.241449	H	-1.41714	3.517635	0.290852
C	-3.61417	-2.16962	1.743346	C	-3.80262	0.123364	2.552929	C	-3.80827	0.148285	2.514059	C	-3.76892	0.241642	2.588416	C	-3.71105	0.08773	2.557304
H	-4.4195	-1.9093	2.417055	H	-4.60803	0.830193	2.702341	H	-4.62362	0.846098	2.650387	H	-4.50261	1.015162	2.770588	H	-4.52394	0.784375	2.709892
C	-3.76749	-2.72387	0.455953	C	-3.92848	-1.16445	1.989311	C	-3.91218	-1.14225	1.951521	C	-4.03402	-1.03519	2.045856	C	-3.82028	-1.19243	1.970129
H	-4.70903	-2.94883	-0.0251	H	-4.83024	-1.60847	1.591686	H	-4.80203	-1.59426	1.535822	H	-4.98766	-1.3997	1.692708	H	-4.72163	-1.64465	1.582623
C	-2.47832	-2.91454	-0.09993	C	-2.63868	-1.75184	1.948948	C	-2.61662	-1.71189	1.929801	C	-2.80845	-1.73212	1.957591	C	-2.5211	-1.76243	1.917588
H	-2.25749	-3.31472	-1.08155	H	-2.41175	-2.73068	1.548305	H	-2.37221	-2.68792	1.533136	H	-2.68239	-2.73208	1.565918	H	-2.27857	-2.72952	1.499107
C	-1.52882	-2.50873	0.867723	C	-1.71877	-0.82471	2.491341	C	-1.71308	-0.77257	2.488533	C	-1.78296	-0.88265	2.462261	C	-1.61534	-0.83213	2.475539
H	-0.45524	-2.56206	0.757844	H	-0.65416	-0.9762	2.611691	H	-0.64887	-0.91217	2.625306	H	-0.73369	-1.1326	2.540913	H	-0.54772	-0.96691	2.583216
C	-2.22314	-2.03022	1.999915	C	-2.43241	0.346305	2.847977	C	-2.44469	0.386866	2.835682	C	-2.3706	0.341446	2.838398	C	-2.34422	0.321907	2.853256
H	-1.77648	-1.63222	2.902207	H	-2.00878	1.242237	3.286101	H	-2.03934	1.288008	3.280003	H	-1.85486	1.199891	3.251143	H	-1.92953	1.22142	3.290912
C	-0.58469	1.893353	-1.17768	C	-0.64531	0.295306	-2.12268	C	-0.69728	0.238691	-2.14263	C	-0.66537	0.188192	-2.12835	C	-0.62497	0.275275	-2.22691
H	-0.88049	2.910402	-1.40162	H	-0.96749	0.751221	-3.04928	H	-1.03661	0.680519	-3.06984	H	-0.97989	0.617935	-3.07043	H	-0.94148	0.747705	-3.14748
C	-0.63205	0.796423	-2.07552	C	-0.76931	-1.07692	-1.78642	C	-0.8256	-1.12568	-1.7781	C	-0.78892	-1.17588	-1.75376	C	-0.73522	-1.11052	-1.92137
H	-0.96149	0.842764	-3.10389	H	-1.21215	-1.83993	-2.41101	H	-1.29686	-1.89425	-2.37411	H	-1.22916	-1.9565	-2.35871	H	-1.15529	-1.86531	-2.57275
C	-0.16832	-0.35151	-1.40783	C	-0.23056	-1.26991	-0.503	C	-0.26127	-1.29778	-0.50365	C	-0.25511	-1.3304	-0.46451	C	-0.2153	-1.32578	-0.63902
H	-0.10648	-1.35079	-1.81465	H	-0.21124	-2.19844	0.047953	H	-0.23692	-2.2163	0.063501	H	-0.23652	-2.24302	0.113666	H	-0.1936	-2.26409	-0.10541

C	0.203488	0.042133	-0.08205	C	0.240359	-0.00701	-0.03205	C	0.240687	-0.03238	-0.07024	C	0.215526	-0.05309	-0.0289	C	0.238609	-0.06695	-0.13189
H	0.425434	-0.62331	0.743184	H	0.541687	0.215974	0.986037	H	0.566611	0.20488	0.93621	H	0.531969	0.194766	0.978022	H	0.475182	0.140611	0.904102
C	-0.05863	1.434214	0.044939	C	-0.02337	0.957608	-1.04877	C	-0.04086	0.914601	-1.09572	C	-0.03853	0.878601	-1.07243	C	-0.03529	0.919919	-1.13147
H	0.093191	2.020516	0.938112	H	0.198724	2.012528	-0.99438	H	0.196728	1.967264	-1.06952	H	0.184468	1.934409	-1.04803	H	0.172681	1.974679	-1.04667
C	-4.41893	-1.13024	-2.19483	C	-4.95827	-3.03258	-0.67713	C	-5.03328	-3.04916	-0.63681	C	-5.03174	-2.9865	-0.5654	C	-4.89921	-3.04686	-0.70143
H	-4.33066	-2.20196	-2.30747	H	-5.03891	-4.02487	-0.2437	H	-5.13657	-4.04539	-0.21689	H	-5.1141	-3.94357	-0.06055	H	-4.99235	-4.05065	-0.30022
C	-5.3539	-0.4484	-1.37396	C	-5.96373	-2.02841	-0.64477	C	-6.03182	-2.03195	-0.61938	C	-6.0124	-1.97891	-0.59547	C	-5.92476	-2.03566	-0.66491
H	-6.11568	-0.90626	-0.75522	H	-6.94438	-2.12747	-0.18864	H	-7.02596	-2.12747	-0.19222	H	-6.98858	-2.02292	-0.12285	H	-6.91565	-2.16107	-0.24045
C	-5.12888	0.939303	-1.52507	C	-5.4606	-0.87896	-1.26312	C	-5.50137	-0.88469	-1.20966	C	-5.49369	-0.87675	-1.3144	C	-5.42328	-0.87957	-1.23535
H	-5.69408	1.72256	-1.04121	H	-5.99749	0.047461	-1.42894	H	-6.02388	0.049812	-1.37745	H	-6.01905	0.044235	-1.53673	H	-5.95203	0.053377	-1.37796
C	-3.60294	-0.16905	-2.81996	C	-3.83116	-2.5092	-1.31787	C	-3.88763	-2.5315	-1.24041	C	-3.89522	-2.52026	-1.26591	C	-3.77276	-2.50961	-1.29697
H	-2.7943	-0.38241	-3.50479	H	-2.90862	-3.041	-1.5189	H	-2.96268	-3.06865	-1.41556	H	-2.98386	-3.07879	-1.44018	H	-2.83413	-3.01651	-1.47585
Ce	3.058549	-0.00309	-0.01113	Nd	3.109053	-0.07546	-0.00576	Sm	3.140091	-0.07963	-0.0017	Eu	3.13649	-0.07183	0.00007	Gd	3.052945	-0.06249	-0.01577
Ce	-2.8327	0.00141	-0.005	Nd	-2.58776	0.289444	0.054459	Sm	-2.55459	0.311148	0.06071	Eu	-2.6129	0.273047	0.039266	Gd	-2.56287	0.288057	0.06966
C	-4.03186	1.118928	-2.3974	C	-4.1147	-1.14872	-1.67772	C	-4.14132	-1.15841	-1.58298	C	-4.17419	-1.20409	-1.73538	C	-4.05639	-1.13203	-1.6289
H	-3.60119	2.065839	-2.69796	H	-3.58897	-0.59251	-2.45354	H	-3.61016	-0.6176	-2.36606	H	-3.60059	-0.66528	-2.48515	H	-3.5406	-0.57723	-2.40963
Tb				Dy				U				Np				Pu			
C	4.183132	-2.50043	0.539897	C	4.1779	-2.50699	0.538192	C	4.079903	-2.57161	0.667775	C	4.241128	-2.48608	0.661569	C	4.179788	-2.53005	0.552839
H	4.91717	-2.80558	-0.19116	H	4.914351	-2.83793	-0.18144	H	4.849798	-2.93646	0.000929	H	4.962554	-2.84463	-0.0601	H	4.927659	-2.85064	-0.15991
C	2.795021	-2.74964	0.481167	C	2.788934	-2.75827	0.484479	C	2.68914	-2.79827	0.533401	C	2.85447	-2.76732	0.660566	C	2.787534	-2.78133	0.469895
H	2.268822	-3.26792	-0.30778	H	2.265528	-3.30778	-0.28686	H	2.206748	-3.35758	-0.25776	H	2.325788	-3.37663	-0.06132	H	2.280332	-3.31672	-0.32151
C	2.198962	-2.15163	1.623584	C	2.195424	-2.15851	1.628228	C	2.035878	-2.16683	1.625211	C	2.277131	-2.10833	1.780542	C	2.171197	-2.19604	1.605707
H	1.147739	-2.17075	1.871657	H	1.146902	-2.1957	1.891727	H	0.97142	-2.17157	1.822126	H	1.235754	-2.14458	2.073189	H	1.116143	-2.2229	1.840095
C	3.216295	-1.53654	2.378995	C	3.214909	-1.53859	2.37828	C	3.021058	-1.54754	2.426635	C	3.304529	-1.41861	2.460019	C	3.176079	-1.575	2.379255
H	3.076268	-0.97387	3.290278	H	3.079908	-0.99001	3.300285	H	2.841377	-0.98665	3.333575	H	3.182637	-0.81554	3.349497	H	3.022502	-1.02968	3.300038
C	4.443753	-1.72533	1.697468	C	4.441681	-1.72868	1.693569	C	4.285893	-1.783	1.829714	C	4.52044	-1.63791	1.761341	C	4.419943	-1.77004	1.725312
H	5.411988	-1.35818	2.011286	H	5.415054	-1.38094	2.017841	H	5.243227	-1.45306	2.214696	H	5.493934	-1.25248	2.037565	H	5.384301	-1.41958	2.073056
C	3.72618	-1.34891	-2.34871	C	3.706967	-1.35017	-2.35741	C	3.547769	-1.39112	-2.41502	C	3.722735	-1.4533	-2.33516	C	3.708233	-1.31011	-2.40103
H	3.729568	-2.42778	-2.29838	H	3.701999	-2.43132	-2.33012	H	3.336369	-2.45232	-2.40839	H	3.701154	-2.53172	-2.25217	H	3.719236	-2.39104	-2.37101
C	2.609802	-0.54476	-2.64835	C	2.604345	-0.53013	-2.6677	C	2.635066	-0.36495	-2.75254	C	2.636062	-0.63222	-2.70588	C	2.58826	-0.50373	-2.6967

H	1.608224	-0.89112	-2.85654	H	1.605221	-0.86419	-2.91159	H	1.600687	-0.49933	-3.03734	H	1.63485	-0.9644	-2.9444	H	1.588288	-0.85135	-2.91532
C	3.014044	0.814198	-2.56512	C	3.025485	0.823716	-2.57735	C	3.304446	0.879007	-2.62008	C	3.076977	0.718443	-2.67663	C	2.989796	0.853387	-2.60131
H	2.370488	1.668962	-2.71369	H	2.401755	1.688911	-2.75816	H	2.870869	1.852466	-2.80919	H	2.471974	1.58557	-2.90788	H	2.350333	1.711	-2.75959
C	4.378463	0.842368	-2.20663	C	4.388147	0.833033	-2.20641	C	4.632627	0.619945	-2.20058	C	4.438303	0.723876	-2.29012	C	4.362306	0.882432	-2.2502
H	4.974578	1.727941	-2.0402	H	5.002921	1.711153	-2.05975	H	5.401795	1.361096	-2.02684	H	5.062897	1.599934	-2.17782	H	4.962962	1.770307	-2.1033
C	4.817489	-0.496	-2.04313	C	4.808409	-0.51219	-2.04173	C	4.781618	-0.78467	-2.06295	C	4.837623	-0.61618	-2.06097	C	4.806325	-0.45718	-2.11028
H	5.812113	-0.8087	-1.75279	H	5.805137	-0.83975	-1.77093	H	5.68752	-1.30236	-1.77166	H	5.824654	-0.9447	-1.75911	H	5.809668	-0.77406	-1.85093
C	2.604725	1.646978	2.122218	C	2.630264	1.655896	2.132999	C	2.800907	1.724639	2.18188	C	2.737755	1.813437	2.072913	C	2.56773	1.655034	2.167204
H	2.000869	1.247329	2.92531	H	2.032863	1.278337	2.953011	H	2.261437	1.358507	3.045553	H	2.173916	1.484344	2.936584	H	2.007735	1.230075	2.98969
C	2.121287	2.363743	0.998331	C	2.141917	2.377281	1.013376	C	2.240299	2.446299	1.098737	C	2.209921	2.485437	0.94003	C	2.019109	2.357237	1.066566
H	1.085937	2.612044	0.810399	H	1.107908	2.647317	0.842607	H	1.199268	2.725674	1.0007	H	1.171634	2.755368	0.795356	H	0.967209	2.553183	0.907745
C	3.218935	2.685408	0.174277	C	3.235408	2.693843	0.180674	C	3.277763	2.752525	0.186496	C	3.272454	2.745284	0.046249	C	3.079116	2.739865	0.214864
H	3.171145	3.197891	-0.77556	H	3.186089	3.22863	-0.75824	H	3.169464	3.29365	-0.74406	H	3.190331	3.233125	-0.91599	H	2.982966	3.270449	-0.72267
C	4.387842	2.157616	0.779709	C	4.407458	2.160892	0.778063	C	4.486394	2.221981	0.707618	C	4.466387	2.239087	0.627584	C	4.293476	2.285051	0.794893
H	5.394612	2.225227	0.390151	H	5.416518	2.250386	0.395018	H	5.467162	2.312172	0.258186	H	5.458833	2.297501	0.199284	H	5.287073	2.435849	0.391284
C	4.006485	1.532791	1.992627	C	4.031736	1.535651	1.993138	C	4.191385	1.589907	1.943189	C	4.134608	1.673692	1.881824	C	3.976472	1.619604	2.005406
H	4.669355	1.025252	2.678154	H	4.701207	1.046825	2.688174	H	4.903777	1.101923	2.595667	H	4.826236	1.209366	2.571865	H	4.682822	1.16178	2.685447
C	-3.37804	2.796971	0.788516	C	-3.42491	2.786107	0.792792	C	-3.69985	2.771586	0.760698	C	-3.52886	2.787115	0.754004	C	-3.47838	2.773583	0.813254
H	-3.39308	2.950135	1.857621	H	-3.45824	2.956584	1.860373	H	-3.66715	2.993102	1.818983	H	-3.59086	2.962614	1.819293	H	-3.51767	2.921422	1.883494
C	-4.418	2.23983	0.012937	C	-4.45992	2.23352	0.0059	C	-4.74648	2.099041	0.084436	C	-4.55056	2.244804	-0.05974	C	-4.5119	2.237778	0.010351
H	-5.36205	1.865281	0.381611	H	-5.42106	1.887898	0.362872	H	-5.64505	1.697285	0.533965	H	-5.5291	1.921973	0.271048	H	-5.47674	1.890752	0.354661
C	-3.97394	2.186204	-1.33524	C	-4.00765	2.192024	-1.34066	C	-4.4049	2.032212	-1.29141	C	-4.0581	2.182493	-1.39201	C	-4.04617	2.20656	-1.33245
H	-4.53168	1.784682	-2.16953	H	-4.56927	1.817598	-2.18682	H	-4.99845	1.574413	-2.07193	H	-4.59859	1.81257	-2.2537	H	-4.59753	1.835813	-2.18646
C	-2.66589	2.706247	-1.3852	C	-2.69891	2.713072	-1.37828	C	-3.14799	2.654609	-1.46199	C	-2.73626	2.676785	-1.39188	C	-2.73013	2.712108	-1.35007
H	-2.0443	2.774542	-2.26626	H	-2.07799	2.807829	-2.25877	H	-2.61552	2.766364	-2.39689	H	-2.08766	2.752498	-2.25448	H	-2.09665	2.801755	-2.22187
C	-2.28215	3.062003	-0.06777	C	-2.32327	3.059377	-0.05501	C	-2.70242	3.102076	-0.19146	C	-2.39816	3.036602	-0.06042	C	-2.36518	3.044907	-0.01805
H	-1.33069	3.48303	0.231026	H	-1.38113	3.500001	0.249052	H	-1.78095	3.634935	0.010729	H	-1.45734	3.459789	0.269876	H	-1.41952	3.465485	0.301024
C	-3.68211	0.155733	2.5029	C	-3.70421	0.115734	2.506692	C	-3.85426	-0.13105	2.488962	C	-3.81832	0.143863	2.550209	C	-3.74104	0.092856	2.545351
H	-4.48253	0.868584	2.637921	H	-4.5346	0.791894	2.662155	H	-4.82241	0.34482	2.577342	H	-4.64821	0.82733	2.673292	H	-4.57324	0.770049	2.684111
C	-3.81231	-1.12708	1.932203	C	-3.78682	-1.17486	1.942111	C	-3.6112	-1.42645	1.974049	C	-3.90217	-1.17521	2.052836	C	-3.82292	-1.21656	2.021786

H	-4.71192	-1.55773	1.518229	H	-4.67762	-1.65383	1.559889	H	-4.35036	-2.10058	1.563909	H	-4.79296	-1.67054	1.692884	H	-4.71239	-1.70653	1.652023
C	-2.52358	-1.71691	1.886001	C	-2.47638	-1.71725	1.898153	C	-2.21331	-1.65855	2.016709	C	-2.59146	-1.72003	2.032312	C	-2.50897	-1.74709	1.972398
H	-2.30016	-2.68485	1.461658	H	-2.21776	-2.69203	1.50673	H	-1.7164	-2.56331	1.691097	H	-2.32927	-2.71284	1.690741	H	-2.24246	-2.72697	1.599221
C	-1.60132	-0.79435	2.426941	C	-1.58851	-0.75843	2.437428	C	-1.59276	-0.50565	2.560735	C	-1.70319	-0.73508	2.528079	C	-1.62033	-0.76636	2.482401
H	-0.53571	-0.94012	2.53068	H	-0.52025	-0.87327	2.56327	H	-0.53565	-0.38129	2.760145	H	-0.63427	-0.84612	2.654455	H	-0.54917	-0.86941	2.592229
C	-2.31014	0.378306	2.787088	C	-2.34102	0.387929	2.794233	C	-2.6066	0.444531	2.844353	C	-2.45415	0.425666	2.832681	C	-2.3771	0.378036	2.823992
H	-1.87947	1.276797	3.209781	H	-1.94645	1.295087	3.236271	H	-2.4524	1.423434	3.281358	H	-2.05969	1.353488	3.228654	H	-1.98452	1.303276	3.227154
C	-0.70716	0.219591	-2.2145	C	-0.70674	0.257619	-2.19591	C	-0.70842	0.5411	-2.17802	C	-0.67864	0.174188	-2.12195	C	-0.75537	0.217459	-2.22023
H	-1.05754	0.663821	-3.13593	H	-1.05822	0.712182	-3.11268	H	-1.04385	1.06381	-3.06331	H	-1.00082	0.578522	-3.07239	H	-1.10832	0.683216	-3.13047
C	-0.83887	-1.14697	-1.85014	C	-0.82028	-1.11733	-1.85547	C	-0.79446	-0.85701	-1.96083	C	-0.77403	-1.18405	-1.72109	C	-0.87655	-1.1565	-1.89083
H	-1.31455	-1.91223	-2.44655	H	-1.28506	-1.87881	-2.46649	H	-1.21949	-1.57586	-2.6475	H	-1.19042	-1.986	-2.31418	H	-1.35236	-1.90982	-2.50241
C	-0.27328	-1.32393	-0.58025	C	-0.23584	-1.31287	-0.59658	C	-0.23808	-1.14815	-0.70091	C	-0.24113	-1.30551	-0.42595	C	-0.30805	-1.35904	-0.62155
H	-0.26336	-2.23914	-0.00965	H	-0.19307	-2.2439	-0.0517	H	-0.17736	-2.12296	-0.24197	H	-0.19992	-2.20759	0.165621	H	-0.27611	-2.29196	-0.07896
C	0.226025	-0.0589	-0.14135	C	0.259504	-0.05127	-0.14169	C	0.212357	0.082618	-0.12818	C	0.202193	-0.00854	-0.01273	C	0.196764	-0.10331	-0.16071
H	0.493644	0.186989	0.878292	H	0.573367	0.1726	0.87155	H	0.509947	0.227088	0.906204	H	0.487342	0.273832	0.996634	H	0.509757	0.112873	0.854246
C	-0.05909	0.891584	-1.16812	C	-0.05112	0.917668	-1.14539	C	-0.09453	1.128444	-1.0539	C	-0.0746	0.89982	-1.07749	C	-0.09682	0.868753	-1.15925
H	0.171044	1.943848	-1.13219	H	0.187548	1.969301	-1.10513	H	0.115773	2.178972	-0.91916	H	0.127538	1.959614	-1.07255	H	0.13979	1.919823	-1.10622
C	-5.01132	-3.01877	-0.6101	C	-4.97039	-3.04578	-0.63815	C	-4.62928	-3.31712	-0.68957	C	-4.9662	-3.03809	-0.60591	C	-5.01631	-3.02023	-0.57125
H	-5.12387	-4.01887	-0.20226	H	-5.07458	-4.05552	-0.25244	H	-4.56984	-4.33563	-0.31794	H	-5.05117	-4.00767	-0.1251	H	-5.0905	-3.9972	-0.10323
C	-6.00235	-1.99129	-0.57796	C	-5.97472	-2.02944	-0.59049	C	-5.74043	-2.44224	-0.53976	C	-5.9543	-2.02038	-0.60093	C	-6.00899	-2.00645	-0.53683
H	-6.99686	-2.08497	-0.15157	H	-6.97041	-2.14409	-0.17213	H	-6.67265	-2.68219	-0.03714	H	-6.92708	-2.08238	-0.12253	H	-6.9737	-2.07944	-0.04342
C	-5.4643	-0.84345	-1.15221	C	-5.4493	-0.86621	-1.13936	C	-5.41481	-1.21132	-1.11533	C	-5.44951	-0.90871	-1.28892	C	-5.51643	-0.88256	-1.21256
H	-5.97811	0.097971	-1.30472	H	-5.97578	0.070139	-1.2789	H	-6.06636	-0.34944	-1.19568	H	-5.98183	0.011443	-1.49633	H	-6.05049	0.042743	-1.39362
C	-3.86447	-2.50551	-1.20721	C	-3.82831	-2.50947	-1.21886	C	-3.61274	-2.63106	-1.35849	C	-3.84438	-2.56353	-1.29716	C	-3.90518	-2.52908	-1.26815
H	-2.94195	-3.04529	-1.38362	H	-2.90135	-3.03854	-1.40445	H	-2.65236	-3.04066	-1.64831	H	-2.93848	-3.12267	-1.49804	H	-2.99933	-3.07998	-1.49142
Tb	3.064962	-0.08101	-0.00416	Dy	3.055549	-0.07653	-0.00518	C	-4.07236	-1.29462	-1.61914	C	-4.12065	-1.22228	-1.72484	C	-4.19225	-1.18139	-1.66972
Tb	-2.51641	0.30105	0.062649	Dy	-2.53284	0.294909	0.057429	H	-3.67449	-0.64957	-2.40476	H	-3.58606	-0.69546	-2.5152	H	-3.68492	-0.65238	-2.4753
C	-4.10062	-1.11949	-1.52551	C	-4.07705	-1.11671	-1.50945	U	3.03488	-0.05988	-0.00129	Np	3.008616	-0.06314	-0.00798	Pu	3.077049	-0.06051	0.006365
H	-3.58879	-0.59415	-2.33179	H	-3.57927	-0.57954	-2.31716	U	-2.60011	0.284831	0.034353	Np	-2.63503	0.217029	0.025397	Pu	-2.58447	0.223212	0.026829

Data arranged in columns.
